# Vagal oxytocin receptors are necessary for esophageal motility and function

**DOI:** 10.1172/jci.insight.190108

**Published:** 2025-05-22

**Authors:** Mohammed Asker, Jean-Philippe Krieger, Ivana Maric, Emre Bedel, Jenny Steen, Stina Börchers, Yuxiang Wen, Francesco Longo, Patrik Aronsson, Michael Winder, Robert P. Doyle, Matthew R. Hayes, Karolina P. Skibicka

**Affiliations:** 1 Department of Physiology/Metabolic Physiology, Institute of Neuroscience and Physiology, The Sahlgrenska Academy at the University of Gothenburg, Sweden.; 2 Wallenberg Centre for Molecular and Translational Medicine, University of Gothenburg, Sweden.; 3 Department of Nutritional Sciences, Pennsylvania State University, University Park, Pennsylvania, USA.; 4 Institute of Veterinary Pharmacology and Toxicology, University of Zurich-Vetsuisse, Switzerland.; 5 Department of Pharmacology, Institute of Neuroscience and Physiology, The Sahlgrenska Academy at the University of Gothenburg, Sweden.; 6 Department of Medical Pharmacology, University of Mersin, Mersin, Turkey.; 7 Department of Chemistry, Syracuse University, Syracuse, New York, USA.; 8 Departments of Medicine and Pharmacology, State University of New York, Upstate Medical University, Syracuse, New York, USA.; 9 Translational Neuroscience Program, Department of Psychiatry, Perelman School of Medicine, University of Pennsylvania, Philadelphia, Pennsylvania, USA.

**Keywords:** Gastroenterology, Neuroscience, Peptides

## Abstract

Oxytocin plays a key role in reproductive physiology but has recently garnered interest for its involvement in modulating feeding behavior. The vagus nerve contributes to feeding behavior control, as well as other gastrointestinal functions. Oxytocin receptors (OTR) are expressed on the vagus, but their role is poorly understood. Herein, we evaluated the contribution of the vagal OTR to food intake and body weight control in male and female rats. Virogenetic knockdown of vagal OTR resulted in reduced body weight and food intake in male rats. Loss of OTR in the vagus also resulted in suppressed locomotor activity in males but hyperactivity in females. Importantly, rats with vagal OTR knockdown, but not controls, exhibited a significantly elevated mortality rate starting 4 weeks after knockdown, with males being disproportionately affected. Mortality followed large eating bouts and was accompanied by abnormal presence of food in the mouth and esophagus, suggesting death by aspiration or food in the airways and suggesting a crucial role of vagal OTR in upper gastrointestinal tract motility. Furthermore, in vivo experiments revealed impaired esophageal transit. Ex vivo findings indicated oxytocin’s contribution to lower esophageal sphincter contraction. Our findings demonstrated a critical role for the oxytocin system: essential function of vagal OTR for esophageal transit and swallowing.

## Abstract 2

Our findings demonstrate a key role of vagal OTR for esophageal transit and swallowing and assign a novel functional role to oxytocin. They also advance our understanding of peripheral oxytocin signaling and its potential therapeutic implications for gastrointestinal disorders.

## Graphical abstract

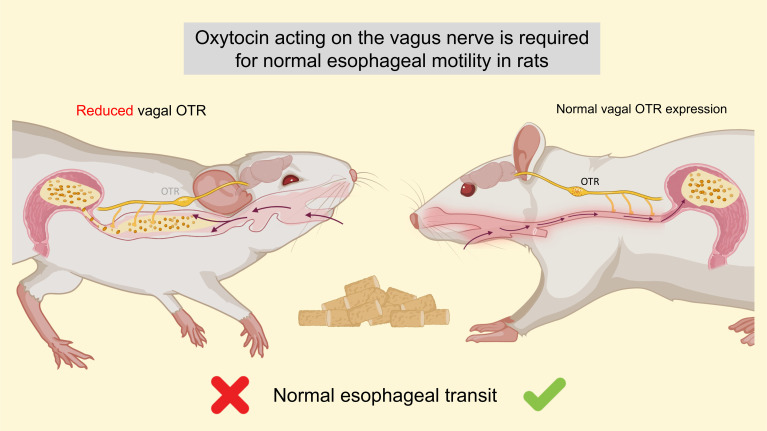

## Introduction

Oxytocin, a multifaceted neuropeptide, is traditionally recognized for its roles in parturition, lactation, and social bonding. However, emerging research suggests oxytocin has a key influence on metabolic processes, particularly in the control of food intake and energy expenditure. Clinical research suggests that oxytocin reduces food consumption (1–4). It acts as a potent short-term regulator of food intake primarily by decreasing meal size and amplifying within-meal satiety signals (5, 6). Peripheral (7–13) and central (7, 8, 14, 15) administration of oxytocin reduces food intake in rodents. Central oxytocin receptors (OTR), found in key brain regions such as the hypothalamus and hindbrain, play a role in energy balance control (16–18). However, peripherally restricted administration of oxytocin reduces food intake and body weight in rodents, without eliciting CNS-mediated effects such as taste avoidance (19). Moreover, peripheral administration of a non-BBB penetrant OTR antagonist elicits hyperphagia and body weight increase in chow-fed rats (20). These finding highlight that peripherally acting oxytocin is sufficient and necessary for body weight control. However, the specific peripheral OTR populations underlying the hypophagic effect remain unknown.

The vagus nerve is a critical neuroanatomical relay, conveying a variety of satiety-related signals from the periphery to the brain. These signals include sensory information from the gastrointestinal tract (GIT) related to stomach distension, nutrient sensing, and satiety (21). Vagal afferent neurons express oxytocin mRNA or protein in fetal rats of both sexes (22), mice of both sexes (23–26), and adult male (27) and female (22) rats, potentially facilitating transduction of the oxytocin signal to the brain. Oxytocin and OTR mRNA may also be expressed in the GIT of both male and female humans (28) and mice (22, 29). Additionally, oxytocin peptide can be detected in the human GIT (30). Vagotomy abolishes the anorexigenic effects of peripherally injected low doses of oxytocin, confirming the necessity of an intact vagus nerve for oxytocin hypophagia (11, 31, 32). Furthermore, peripherally injected oxytocin-B12 conjugate, an oxytocin analogue that does not cross the blood-brain barrier (BBB), is similarly potent to native oxytocin, which crosses the BBB, at reducing food intake in rats (19). Here, we aimed to specifically disrupt OTR signaling in the vagus nerve to investigate whether this intervention is sufficient to disrupt food intake and body weight control in male and female rats.

The vagus nerve also provides robust afferent innervation to the mucosa throughout the mouse esophagus, potentially contributing to sensing intraluminal content and to regulation of swallowing behavior, a complex and fundamental process for ingestive behavior (33). Additionally, there is an abundance of vagal afferent terminals on myenteric neurons in the rat esophagus and smooth muscle layers at the esophago-gastric junction (34). The dorsal vagal complex plays a crucial role in reflex relaxation of the lower esophageal sphincter (LES), mediated by vagal sensory and motor neurons (35, 36). Esophageal motility disorders can result in a wide range of pathologies ranging from sustained contractions (37) to a failure to relax (38, 39) that affect either esophageal sphincters and smooth muscles. Notably, cumulative evidence suggests that vagal afferent terminals are a possible target for pharmacologic intervention for the treatment of gastrointestinal disorders such as gastroesophageal reflux disease. Nevertheless, it is not yet clear what specific targets within vagal neurons is or are responsible for regulating esophageal motility. However, previous reports have linked oxytocin to swallowing behavior regulation with possible benefits for esophageal obstruction management (40, 41) but without evidence for a role of any specific OTR population in this process.

Vagus nerve also plays an important role in thermoregulatory control (42–46); with studies reporting both hyperthermic and hypothermic effects after vagal nerve stimulation. A vagally mediated regulation of body temperature or brown adipose tissue (BAT) thermogenesis was reported for ghrelin (47) and glucagon-like peptide-1 in rats (48). Oxytocin also increases energy expenditure mainly by activating BAT and inducing white adipose tissue (WAT) beiging (49). OTR-deficient mice have lipid accumulation in BAT in addition to impaired cold-stimulated thermogenesis (50). Similarly, mice lacking oxytocin show late-onset obesity without an increase in food intake, indicative of reduced metabolism (51). Peripheral, but brain penetrant, oxytocin administration produces an initial hypothermic response followed by a modest hyperthermic response (52). Moreover, we have previously shown that peripherally restricted oxytocin induces similar thermoregulatory dynamics to BBB-penetrant oxytocin (19). Given that our study suggested a peripheral site of action for the thermoregulatory actions of oxytocin, we also determined whether vagal OTR contribute to body temperature regulation.

The overarching aim of the current set of studies is to elucidate the contribution of the vagal OTR to the control of feeding behavior, thermogenesis, and gastrointestinal motility. To reduce vagal OTR, we employed a virogenetic knockdown (Kd) technique targeted to the nodose ganglia of male and female rats (6, 20, 53). Following the selective disruption of the vagal oxytocin signaling, feeding behavior, body weight, and thermogenesis and locomotion were measured over a 2-month period. Moreover, gastric emptying and duodenal, stomach, and esophageal food content were also measured in a terminal refeeding experiment. Ex vivo pharmacological studies assessed the contribution of oxytocin to LES contraction. Molecular experiments were performed to link OTR-expressing nodose neurons with well-established esophageal motility markers. Experiments were conducted in male and female rats, as most previous studies overlooked assessing the role or presence of OTR in female vagus nerve (11, 22, 26, 31, 32), and sex differences are known to exist in the central and peripheral oxytocin systems (54).

## Results

### OTR Kd in nodose ganglia results in sex-specific effects on body weight, food intake, and mortality rates.

**Figure 1:**
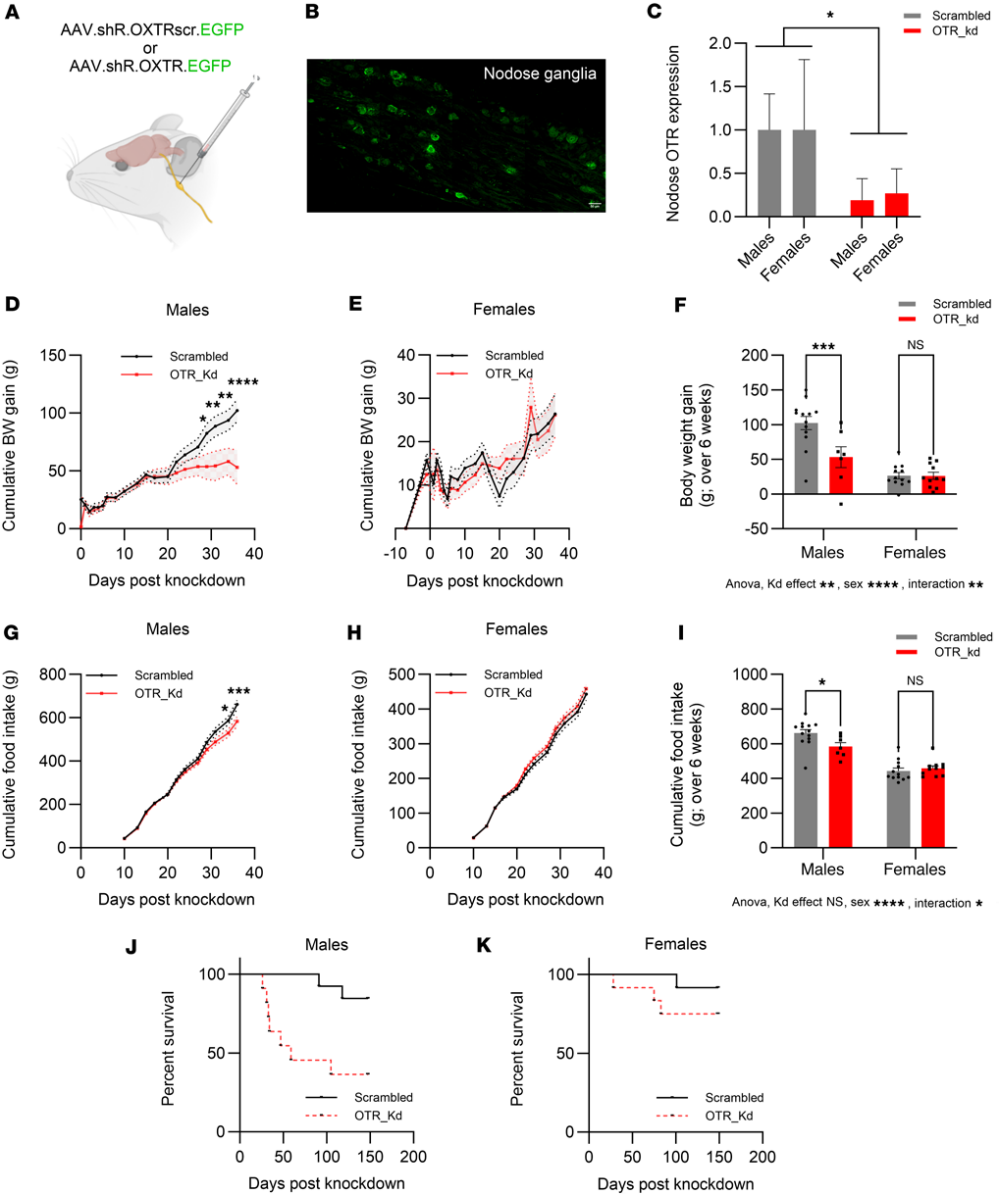
OTR knockdown in nodose ganglia affects body weight, food intake, and mortality in rats, in a sex-specific manner. (**A**) Diagram of the experimental design showing nodose ganglia injections with either scrambled (AAV.shR.OXTRscr.EGFP) or OTR knockdown (AAV.shR.OXTR.EGFP) viruses. (**B**) Confocal microscopy image showing EGFP-labeled viral expression in the nodose ganglia. Scale bar: 50 μm. (**C**) qPCR analysis confirms a significant reduction in OTR expression in the nodose ganglia after injection, normalized to GAPDH. (**D**–**F**) Nodose OTR knockdown results in a significant reduction in body weight gain in males but not in females. (**G**–**I**) OTR knockdown also significantly reduced food intake in males but not in females. (**J** and **K**) Kaplan-Meier survival analysis shows increased mortality in males with OTR knockdown compared with controls, whereas females were less affected by the nodose OTR loss. Data are shown as mean ± SEM and analyzed by 2-way ANOVA and post hoc Holm-Šidák tests unless otherwise stated; *n* = 11–12 for females and 7–13 for males. *P < 0.05, **P < 0.01, ***P < 0.001, ****P < 0.0001.

To validate the OTR Kd model, viral expression within nodose ganglia marked by EGFP label expression was visualized with confocal microscopy (Figure 1, A and B). Moreover, quantitative PCR (qPCR) was utilized to measure OTR expression relative to GAPDH as the housekeeping gene (Figure 1C). A significant reduction in OTR expression in the nodose ganglia injected with AAV1 U6.shR.OXTRscr.CB7.EGFP.SV40 was found in both sexes. There were no significant differences in the Kd degree between sexes. This change was specific to OTR, as no changes in expression of other genes — *Actb* or *Igf-1*, presumed unrelated to OTR signaling — were identified (Supplemental Figure 3, A–F; supplemental material available online with this article; https://doi.org/10.1172/jci.insight.190108DS1).

Body weight and food intake were measured for 6 weeks following viral infusion. Knocking down OTR in the nodose ganglia resulted in a sex-divergent effect on body weight gain, where body weight was reduced in males but not in females (Figure 1, D–F). Body weight in males started diverging about 3 weeks after Kd, timing consistent with peak of the Kd effect. Two-way ANOVA indicated a significant effect of Kd [F_(1,39)_ = 8.246; *P* = 0.006] and sex [F_(1,39)_ = 35.82; *P* = 0.0001], and the interaction of sex and Kd [F_(1,39)_ = 8.097; *P* = 0.007]. Post hoc tests indicated a significant effect of Kd in males (*P* = 0.0009) but no effect in females (*P* = 0.99).

The OTR Kd also resulted in sex divergent changes in food intake, where males, but not females, displayed a significant reduction in food intake (Figure 1, G–I), with the reduction in feeding emerging with a similar timeline as the body weight gain reduction. Two-way ANOVA indicated a nonsignificant effect of Kd [F_(1,39)_ = 2.503; *P* = 0.12] while results were significant for sex [F_(1,39)_ = 76.01; *P* = 0.0001], and the interaction of sex and Kd [F_(1,39)_ = 5.604; *P* = 0.02]. Post hoc tests indicate a significant effect of Kd in males (*P* = 0.02) and not in females (*P* = 0.8).

Food intake was significantly reduced upon refeeding of chow in males at 30 and 60 minutes of refeeding (Supplemental Figure 1, A and C), without any significant differences noted in females. Interestingly intake of palatable liquid food was not alerted in either sex when analyzed separately (Supplemental Figure 1, E and F); however, when both sexes were combined, a significant reduction in food intake was found (Supplemental Figure 1G).

A Kaplan-Meier survival analysis was performed to evaluate the effect of OTR Kd on the survival time. In males, Kaplan-Meier survival curve illustrated a significantly lower survival time for OTR-Kd group in comparison with scrambled group (Figure 1J). The log-rank test indicated a significant difference in survival between the groups (χ²[1] = 7.292, *P* = 0.0069). To further validate the observed differences, a Gehan-Breslow-Wilcoxon test was conducted. This test, which places more emphasis on earlier events, also showed a significant difference in survival between the groups (χ²[1] = 7.848, *P* = 0.0051). In females, although several deaths were noted for the OTR-Kd group, a Kaplan-Meier survival curve suggested a similar survival time for both OTR-Kd and scrambled groups (Figure 1K). The log-rank test displayed no difference in survival between the groups (χ²[1] = 1.306, *P* = 0.2530). The Gehan-Breslow-Wilcoxon test also indicated lack of significant effect between the groups in females (χ²[1] = 1.446, *P* = 0.2291).

### Loss of vagal OTR resulted in sex-divergent effects on cumulative home-cage locomotor activity, without affecting core body temperature.

**Figure 2:**
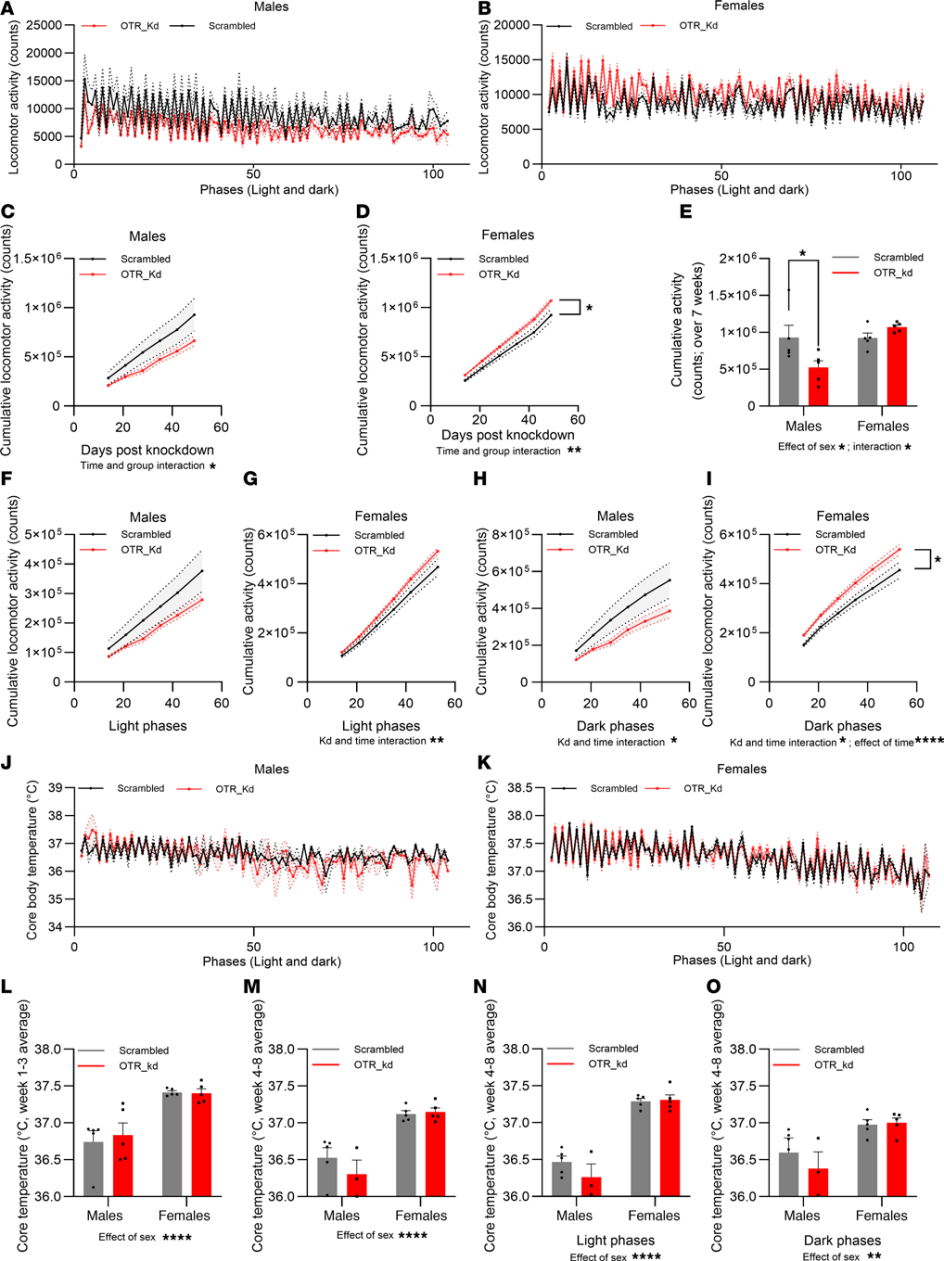
OTR knockdown affects home-cage locomotor activity in a sex-divergent manner, with no concomitant changes in core body temperature. (**A** and **B**) Telemetric home-cage recording of home-cage locomotor activity over 50 days, with cumulative activity during light and dark cycle phases shown in males and females. (**C**–**I**) Cumulative home-cage locomotor activity decreases in males (**C**) and increases in females (**D**) after nodose OTR knockdown. Analysis of cumulative activity at 7 weeks (**E**) confirms these sex differences. No difference in activity was detected during the light phase in males; however, a clear trend for a reduced activity is present (**F**). Females show an increased locomotion after OTR Kd (**G**). Similar changes are identified for dark phase locomotor activity, with reductions detected in males (**H**) and increases in activity found in females (**I**) with OTR knockdown. (**J**–**O**) Core body temperature recordings over 50 days indicate no effect of OTR Kd on this parameter in either sex. However, as expected all females, irrespective of treatment, display higher average body temperatures than males during all phases, analyzed over first 3 weeks (**L**), or latter 4 weeks (**M**) after OTR Kd. Similar differences are also seen segregating temperature data by light (**N**) and dark (**O**) phases. Data are shown as mean ± SEM and analyzed by 2-way ANOVA and post hoc Holm-Šidák; *n* = 3–5 for males and *n* = 5 for females. *P < 0.05, **P < 0.01, ***P < 0.001, ****P < 0.0001.

To investigate whether OTR Kd in nodose ganglia alter core body temperature and locomotor activity, these parameters were telemetrically recorded for 7 weeks after the OTR Kd (Figure 2, A–O). General activity and core temperature fluctuations over the subsequent light and dark phases during the recorded period in males and females are displayed in Figure 2, A, B, J, and K.

Loss of vagal OTR resulted in sex-divergent effects on home-cage locomotor activity (Figure 2, C–I). OTR-Kd males displayed lower cumulative locomotor activity over time (Figure 2C). Two-way ANOVA results were not significant for Kd main effect [F_(1,8)_ = 2.177; *P* = 0.1784] but indicate significant effect of time [F_(0.7652,5.357)_ = 66.98; *P* = 0.0004] and the interaction of time and Kd [F_(6,__42)_ = 2.383; *P* = 0.0452]. Surprisingly, the nodose Kd resulted in hyperlocomotion in females, where OTR-Kd females moved more than the control group (Figure 2D). Two-way ANOVA results indicate significant main effect of Kd [F_(1,8)_ = 5.767; *P* = 0.0431] and time [F_(1,051,__8,408)_ = 670.8; *P* < 0.0001] as well as the interaction of time and Kd [F_(6,48)_ = 3.760; *P* = 0.0038]. Comparing the sum of locomotion at the end of the 7 weeks between groups and sexes further confirmed sex differences (Figure 2E). Two-way ANOVA results were not significant for Kd main effect (F_(1,16)_ = 1.616; *P* = 0.2218) but were significant for sex (F_(1,16)_ = 7.115; *P* = 0.0169) as well as for the interaction of sex and Kd (F_(1,16)_ = 7.474; *P* = 0.0147).

To further understand whether general locomotion differences discovered are circadian rhythm dependent, we sought to analyze cumulative locomotor activity within light or dark phase separately (Figure 2, F–I). During light phases, males’ locomotor activity was similar between groups (Figure 2F). Two-way ANOVA results were not significant for Kd main effect [F_(1,8)_ = 1.582; *P* = 0.2440], and the interaction of time and Kd [F_(6,__42)_ = 1.916; *P* = 0.1005] but were significant for time [F_(0.7859,5.501)_ = 67.39; *P* < 0.0004]. However, female groups showed locomotor differences during light phases with OTR-Kd group moving more over time than control group (Figure 2G). Two-way ANOVA results were not significant for Kd main effect [F_(1,8)_ = 3.085; *P* = 0.1171] while it was significant for time [F_(1,084,8,674)_ = 719.8; *P* < 0.0001], and the interaction of time and Kd [F_(6,48)_ = 3.261; *P* = 0.0090]. In dark phases, OTR-Kd males depicted reduced locomotor activity over time in comparison with control group (Figure 2H). Two-way ANOVA results were not significant for Kd main effect [F_(1,8)_ = 2.574; *P* = 0.1473], while they were significant for time [F_(0.7626,5.338)_ = 63.49; *P* = 0,0005] and the interaction of time and Kd [F_(6,42)_ = 2.59; *P* = 0.0316]. In contrast to male results, female OTR Kd showed higher activity within dark phases compared with control group (Figure 2I). Two-way ANOVA results were significant for Kd main effect [F_(1,8)_ = 7.679; *P* = 0.0243], time [F_(1.044,8.353)_ = 445.6; *P* < 0,0001], and the interaction of time and Kd [F_(6,48)_ = 3.122; *P* = 0.0115].

Core body temperature variations were recorded over 7 weeks in males (Figure 2J) and females (Figure 2K). Core body temperature was not different between groups within each sex when averaged over light and dark phases combined or either phase separately (Figure 2, L–O). However, there were sex differences detected, with females exhibiting higher body temperature than males when averaged over all phases in the first 3 weeks of recording (Figure 2L) — with 2-way ANOVA results significant for sex [F_(1,16)_ = 28.52; *P* < 0.0001] but not significant for Kd [F_(1,16)_ = 0.1245; *P* = 0.7288] or interaction of sex and Kd [F_(1,16)_ = 0.2090; *P* = 0.6537] — and the latter 4 weeks of recording that followed the surgeries (Figure 2M), where 2-way ANOVA results indicate a significant effect of sex [F_(1,14)_ = 44.91; *P* < 0.0001] but not Kd [F_(1,14)_ = 0.8323; *P* = 0.3770] or interaction of sex and Kd [F_(1,14)_ = 1.338, *P* = 0.2667]. A similar effect of sex was detected when temperature was averaged over light phases only (Figure 2N). Two-way ANOVA results indicate a significant effect of sex [F_(1,14)_ = 121.6; *P* < 0.0001] but not Kd [F_(1,14)_ = 1.257; *P* = 0.2811] or interaction of sex and Kd [F_(1,14)_ = 1.755; *P* = 0.2064] or if averaged over dark phases only (Figure 2O). Two-way ANOVA results were significant for sex [F_(1,14)_ = 11.82; *P* = 0.0040] but not significant for Kd [F_(1,14)_ = 0.4342; *P* = 0.5206] or interaction of sex and Kd [F_(1,14)_ = 0.7023; *P* = 0.4161]. Higher temperature detected in females compared with males is consistent with previous literature (55, 56).

### Acoustic startle response (ASR).

No differences were detected in anxiety-like behavior as measured by the ASR test in either sex (Supplemental Figure 2), although OTR-Kd males tended to show reduced ASR responses, consistent with reduced anxiety-like behavior, while females tended to show responses more consistent with an increase in anxiety-like behavior.

### Gastric emptying rate was reduced by exogenous oxytocin but not affected by OTR loss in nodose ganglia.

**Figure 3:**
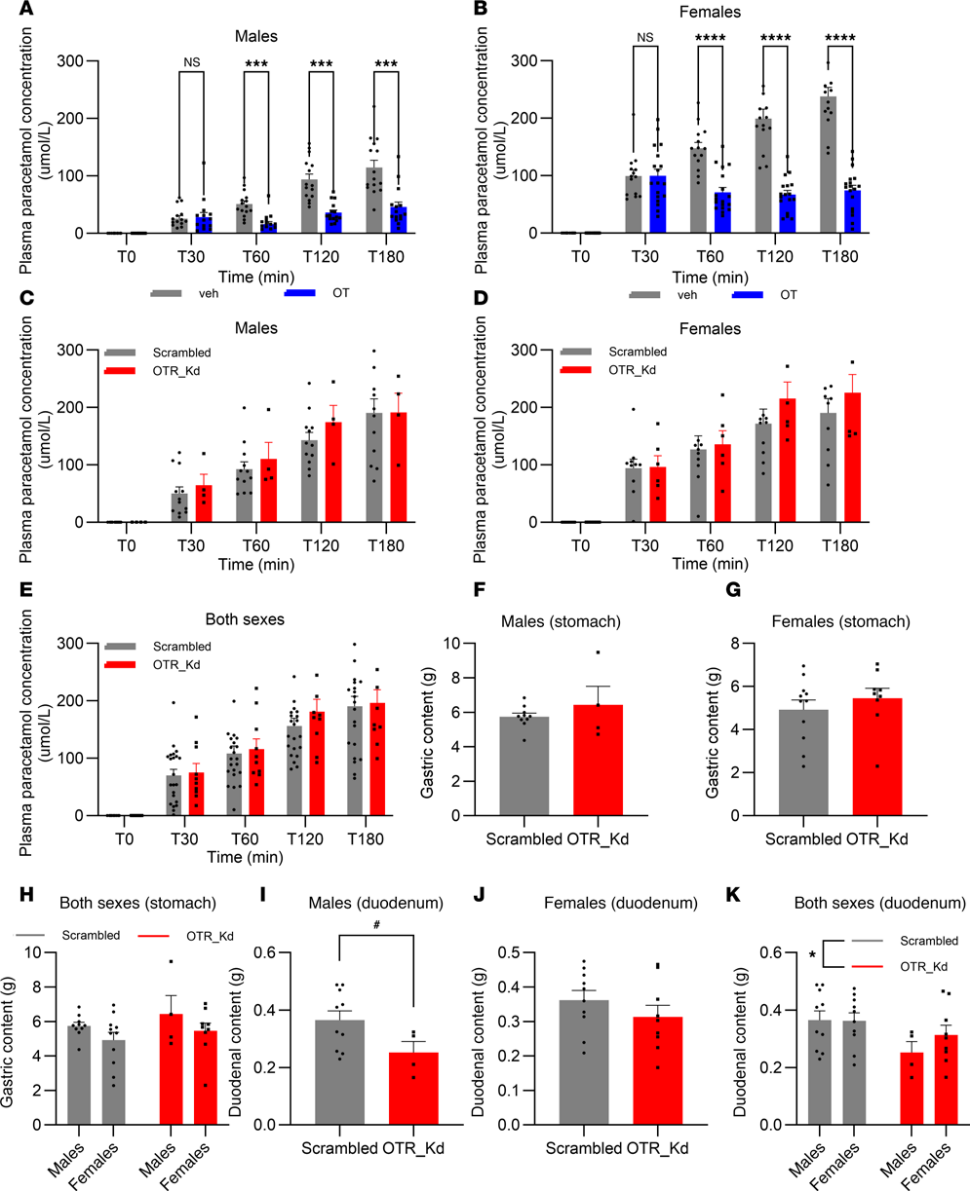
Exogenous oxytocin reduces gastric emptying rate, but OTR knockdown does not affect this parameter. (**A** and **B**) Gastric emptying rate declines significantly following i.p. oxytocin injection in both males (**A**) and females (**B**). (**C**–**E**) OTR knockdown does not affect gastric emptying rate in either sex. (**F**–**H**) Additionally terminal measurements of gastric content weights after a meal show no significant differences between nodose OTR knockdown and controls in either sex. (**I**–**K**) Duodenal content, however, was reduced in OTR knockdown groups. Data are shown as mean ± SEM and analyzed by 2-way ANOVA and post hoc Holm-Šidák (**A**–**E**, **H**, **K**) or 2-tailed *t* tests (**F**, **G**, **I**, **J**) when only 2 groups were compared; *n* = 4–15 for males *n* = 14–18 for females. *P < 0.05, **P < 0.01, ***P < 0.001, ****P < 0.0001, ^#^*P* < 0.1.

Exogenous peripheral oxytocin application (1 mg/kg, i.p.) to rats with intact vagal OTR reduced gastric emptying rate of a chow test meal, as measured by detection of ingested paracetamol concentrations in blood after a test meal; the significant decline in emptying commenced at 60 minutes after meal ingestion in males (Figure 3A). Two-way ANOVA results indicate a significant effect of oxytocin treatment [F_(1,28)_ = 24.31; *P* < 0.0001], and time [F_(1.948,54.54)_ = 58.75; *P* < 0.0001] as well as the interaction of time and treatment [F_(4,112)_ = 15.30; *P* < 0.0001]. Similarly in females (Figure 3B), 2-way ANOVA results indicate a significant effect of oxytocin treatment [F_(1,30)_ = 49.99; *P* < 0.0001], and time [F_(2.687,79.93)_ = 132.5; *P* < 0.0001] as well as the interaction of time and treatment [F_(4,119)_ = 51.79; *P* < 0.0001]. However, gastric emptying rate was not affected by loss of OTR in nodose ganglia male or female rats (Figure 3, C–E). Additionally, in a terminal experiment, contents of stomach and duodenum were measured after refeeding with a test meal. Gastric content weights were not different between the groups in either sex or both combined (Figure 3, F–H). Two-way ANOVA results did not indicate a significant effect of Kd [F_(1,29)_ = 1.452; *P* = 0.2376], but they did indicate a trend for sex [F_(1,30)_ = 3.170; *P* = 0.0851] and no interaction of Kd and sex [F_(1,30)_ = 0.0242; *P* = 0.8774]. Duodenal content was, however, decreased by the loss of nodose OTR (Figure 3, I–K). Two-way ANOVA results indicate a significant effect of Kd [F_(1,29)_ = 5.045; *P* = 0.0325] but not sex [F_(1,29)_ = 0.6599; *P* = 0.4232] or the interaction of Kd and sex [F_(1,29)_ = 0.7854; *P* = 0.3828].

### OTR-Kd results in esophageal food retention.

**Figure 4:**
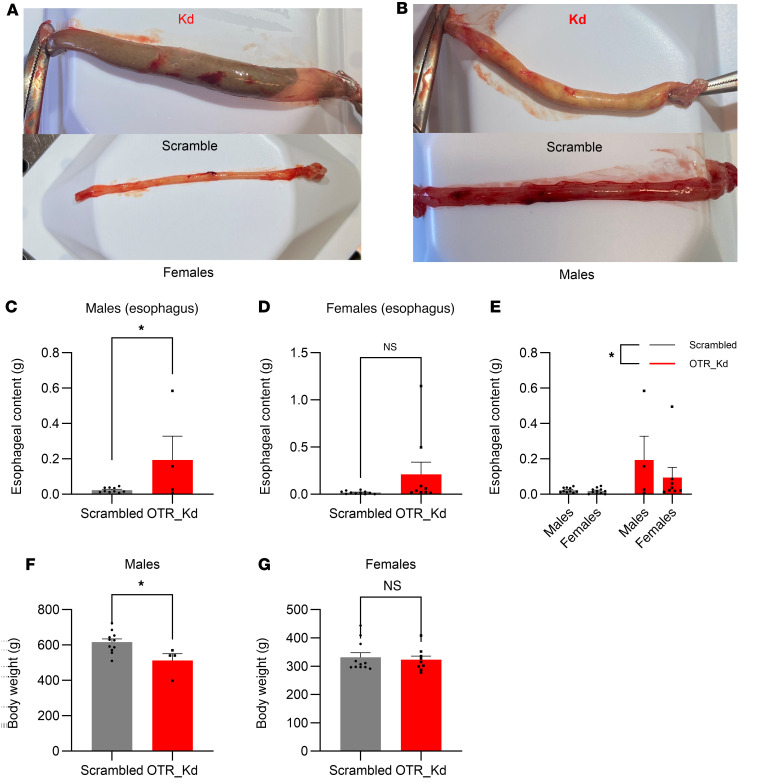
OTR knockdown leads to esophageal food retention. (**A** and **B**) Representative images depict large food mass trapped in the esophagus of OTR knockdown rats compared with controls. (**C**–**E**) Quantification shows significant food retention in the esophagus of OTR knockdown rats. (**F** and **G**) Body weights on the day of terminal experiment are lower in OTR knockdown males but not in females. Data are shown as mean ± SEM and analyzed by 2-way ANOVA and post hoc Holm-Šidák or 2-tailed *t* tests (**C**, **D**, **F**, **G**) when only 2 groups were compared; *n* = 4–11 for males and 9–11 for females. *P < 0.05.

Postmortem analysis suggested that the primary cause of death was food aspiration in the lungs. Loss of vagal OTR affected swallowing behavior in male rats, an effect that was consistently observed exclusively in the Kd group (Supplemental Videos 1–3). Rats with nodose OTR Kd displayed swallowing difficulties (dysphagia), frequent gasping for air, and other reactions consistent with food trapped in the esophagus and airways. This idea was further supported by the terminal experiment where OTR-Kd rats showed a considerable amount of food mass trapped in the esophagus, with the normal rate of evacuation to the stomach obstructed (Figure 4, A and B). OTR males retained, on average, more food in the esophagus compared with controls, but surprisingly food was also retained in the esophagi of the female rats, albeit with a bit more variability (Figure 4, C–E). Two-way ANOVA results indicate a significant effect of the Kd [F_(1,30)_ = 5.151; *P* = 0.0306] but not sex [F_(1,30)_ = 0.0054; *P* = 0.9417] or the interaction of Kd and sex [F_(1,30)_ = 0.0197; *P* = 0.8893]. Grubbs outlier test identified 1 female as an outlier. Her presence does not change the conclusion of the study or the statistical outcome in a meaningful way: if her data are removed from the group, the results remain statistically significant (*P* = 0.0184 after removal and 0.0172 prior). In males, no statistical outliers were identified. In the combined dataset, again only 1 female data point met outlier criteria (value 1.1476), and exclusion of this point strengthened the main effect of the Kd [2-way ANOVA: effect of Kd, F_(1,29)_ = 7.614; *P* = 0.0099]. Supplemental Table 1 includes ΔCt values for OTR expression (average ΔCt is 12.3 versus 14.6 for control and Kd, respectively). We note that the 1 rat in the Kd group with ΔCt of 12.9 (thus highest OTR expression in the group) also had the least amount of food retained (0.019 g) and levels comparable with the level of food in the esophagus of controls. Body weights measured on the day of the terminal experiment continued to indicate lower weights in OTR-Kd males (Figure 4F) but not in females (Figure 4G). Thus, loss of vagal OTR resulted in abnormally trapped food in the esophagus, and this evoked food aspiration in the lungs and death in a sex-dependent manner.

### Colocalization of OTR with Runx3 and Prox2 genes in nodose ganglia and esophagus.

**Figure 5:**
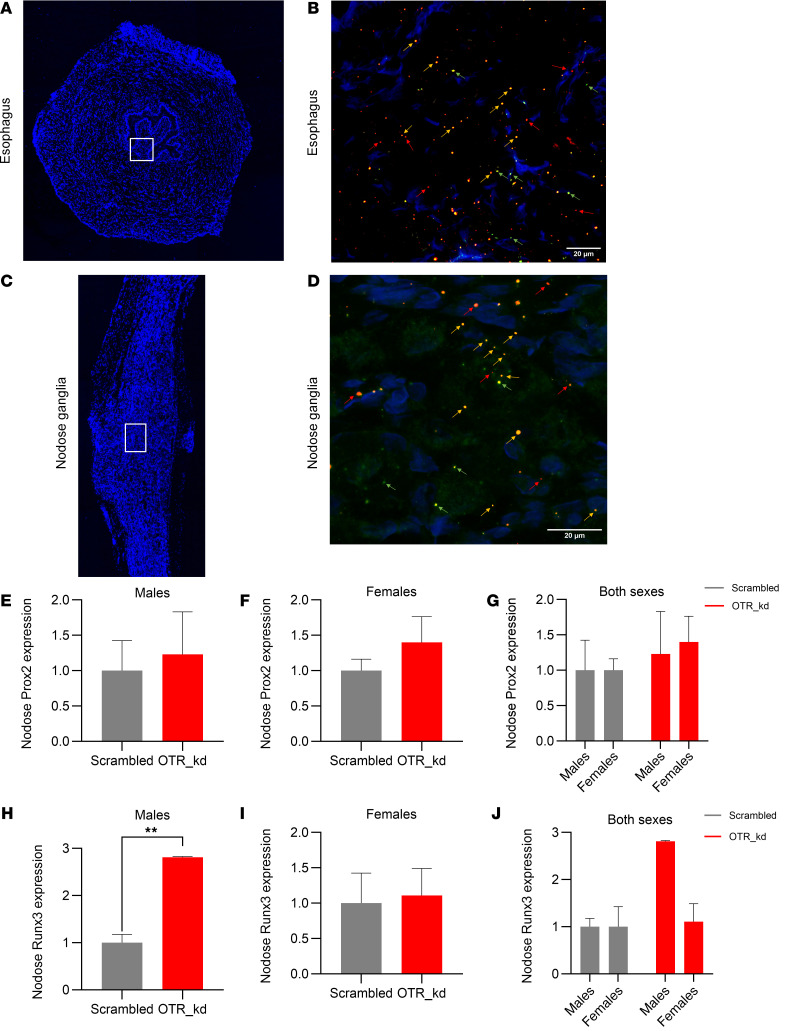
OTR colocalizes with Runx3 and Prox2 in nodose ganglia and esophagus. (**A**–**D**) RNAScope confocal images show colocalization of *Otr* (orange), *Runx3* (red), and *Prox2* (green) in transverse sections of the esophagus (**A** and **B**) and longitudinal sections of nodose ganglia (**C** and **D**). Scale bar: 20 μm (**B** and **D**). (**E**–**G**) qPCR analysis confirms *Prox2* expression in nodose ganglia of rats without sex differences. (**H**–**J**) *Runx3* expression increases in males but not females following *OTR* knockdown. Data are shown as mean ± SEM and analyzed by 2-way ANOVA and post hoc Holm-Šidák or 2-tailed *t* tests when only 2 groups were compared; *n* = 2–3 for males and for females 8–9. *P < 0.05, **P < 0.01.

To further support the idea of nodose OTR signaling association with esophageal motility, RNAScope was utilized to determine whether *Otr* is coexpressed with *Runx3* and *Prox*2, markers previously linked with esophageal motility (23). Transverse section of esophagus (Figure 5A) and longitudinal section of nodose ganglia (Figure 5C) were visualized using confocal imaging. RNAScope confocal images indicate the colocalization of *Otr* (orange signals), *Runx3* (red signals), and *Prox2* (green signals) in the esophagus (Figure 5B) as well as in the nodose ganglia (Figure 5D) with corresponding cellular nuclei of esophagus and nodose ganglia stained with DAPI.

qPCR further confirmed the presence of *Prox2* transcript in nodose ganglia with similar expression levels in males and females (Figure 5, E–G). To determine whether there is a functional relationship between nodose ganglia *Otr* and *Prox2* and *Runx3*, expression of these genes was measured after vagal OTR Kd. Loss of vagal OTR was associated with increased *Runx3* expression in males but not females (Figure 5, H–J).

### Oxytocin and LES contractility ex vivo.

**Figure 6:**
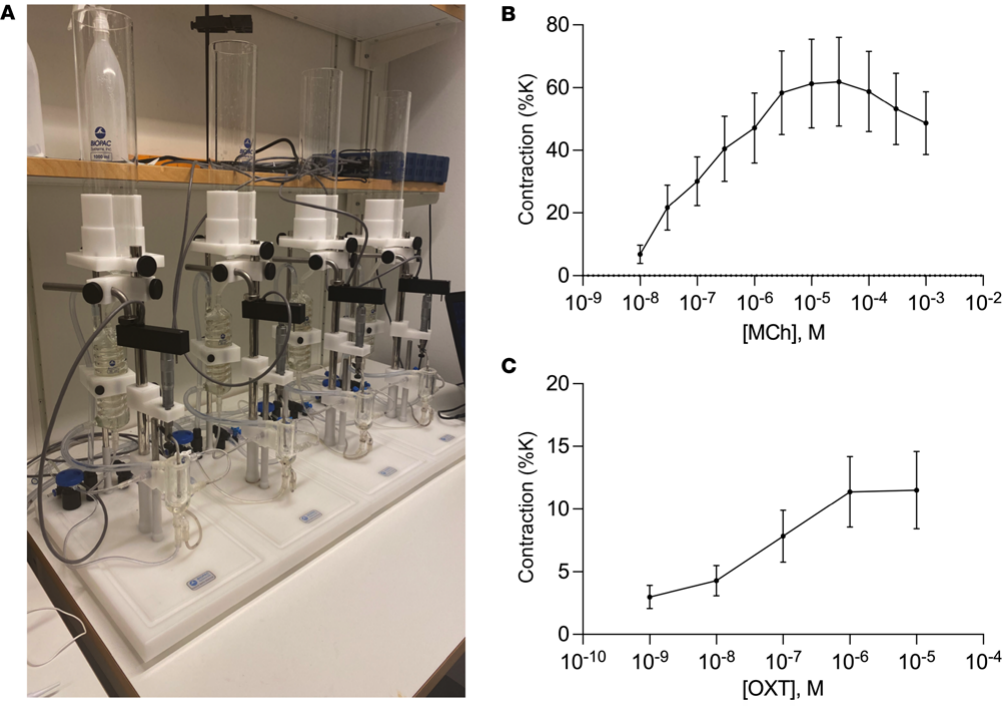
Lower esophageal sphincter (LES) contractile responses to methacholine (MeCh) and oxytocin. (**A**) Organ bath setup for LES contraction recordings. (**B**) Contractile responses to MeCh (1 × 10^–8^M to 1 × 10^–3^M). (**C**) Contractile responses to oxytocin (10^–9^–10^–5^ M). Each response was normalized to the initial high K^+^-induced (maximum) contraction and is therefore shown as percentage of maximum contraction. All tissues responded in a dose-dependent manner to both MeCh and oxytocin (OT), with MeCh inducing comparatively greater contractile responses than oxytocin. Data are shown as mean ± SEM; *n* = 7.

To provide additional evidence of a potential role of oxytocin in esophageal food transit, the LES contractions in response to oxytocin were determined ex vivo. A tissue-bath experiment on isolated LES preparations from rats showed robust contractions to smooth muscle depolarization. The contractile force to potassium (124 mM), a test of tissue viability, was 17.60 ± 4.12 mN. Additionally, cholinergic receptor stimulation with the muscarinic agonist methacholine (1 × 10^–8^M to 1 × 10^–3^M) was used as a positive control and showed a maximal contraction of 10.50 ± 4.09 mN (Figure 6, A and B) at 1 × 10^–5^M, followed by somewhat reduced responses at higher concentrations. Stimulation with oxytocin (1 × 10^–9^M to 1 × 10^–5^M) showed smaller but clearly concentration-dependent contractions, reaching a maximal contraction of 1.50 ± 0.58 mN (Figure 6C) at 1 × 10^–6^M, with a maintained plateau response at 1 × 10^–5^M, suggesting that oxytocin can induce LES contractility and likely plays a modulatory role in this process.

## Discussion

Our study aimed to elucidate the role of vagal oxytocin signaling in feeding behavior and thermogenesis control, as well as esophageal and gastrointestinal motility in male and female rats. Contrary to our initial hypothesis that OTR Kd in the nodose ganglia would interrupt satiety signaling by peripheral oxytocin and consequently lead to increased body weight, OTR Kd instead led to a reduction in cumulative body weight gain and food intake in male, but not female, rats. Loss of OTR in the nodose also resulted in suppression of home-cage locomotor activity in males but rather unexpectedly produced hyperactivity in females. Importantly, males with OTR Kd had high mortality, starting 4 weeks after treatment. Mortality often followed large eating bouts and was accompanied by abnormal presence of food in the mouth and esophagus, suggesting death by aspiration or food in the airways. Terminal experiments revealed an accumulation of food within the esophagus of the OTR Kd rats, indicative of impaired esophageal motility. Moreover, ex vivo studies supported a role of oxytocin in LES contraction. Therefore, our findings demonstrate a novel role for the oxytocin system and a critical role of nodose OTR for esophageal food transit and swallowing. Loss of OTR and selectively in the nodose was accompanied by significant mortality in male rats, leading to the conclusion that vagal oxytocin signaling is necessary for the maintenance of normal esophageal food transit to the extent that its lack affects animals’ survival.

The survival analysis further revealed a sex bias in mortality rate of the vagal OTR Kd. Male rats with OTR Kd had a significantly lower survival rate compared with their control counterparts, while no significant difference in survival was observed in female rats, though there were numerically more deaths in the Kd group. The disproportionate mortality observed in male rats was accompanied by more frequent observations of swallowing difficulties (dysphagia), frequent gasping for air, and sneezing-like reactions in males. Therefore, dysphagia following food buildup in esophagus is likely the primary effect of loss of vagal OTR, while the repeated aspiration incidences of varying severity led to the secondary outcomes including anorexia, body weight loss, hypolocomotion, and death (Supplemental Videos 1–3). Aspiration incidences started around 3 weeks after surgeries, coinciding with the peak of viral Kd expression. Higher mortality followed the dark phase, the circadian phase when rats consume most of their daily food load, or after large eating bouts following overnight fasting. Rats were found with food remains/secretions at the mouth and nose. These unexpected observations led us to pursue the hypothesis that loss of vagal OTR disrupts gastric emptying rate or esophageal motility, leading to dangerous food transit blockade in upper GIT.

Loss of vagal OTR also affected locomotor activity, initially investigated due to its contribution to energy expenditure. Loss of vagal OTR in male rats was associated with reduced cumulative locomotor activity, whereas hyperlocomotion was found in females. Sex differences in locomotion are evident in literature (57–60), and gonadal steroids contribute to sex-typical patterns of movement (61). Sex differences in locomotion in response to oxytocin were also reported. Oxytocin decreased cocaine-triggered movement in females but had no effect in males (62). In a novel environment, females exhibited decreased motor activity at lower oxytocin doses than males (63). Thus, oxytocin seems to be more important for locomotor control in females compared with males, and our finding of enhanced activity in females after loss of vagal OTR might indicate that vagal OTR are indeed necessary for endogenous oxytocin to reduce locomotion, possibly as part of the behavior satiety sequence. While reduced activity in males could be interpreted as an indication that endogenous oxytocin acts on the vagal OTR to increase activity, it is much more likely that the reduced activity is a result of esophageal motility disruption, resulting stress, and the declining health of the male rats. Therefore, we posit that hypolocomotion is secondary to esophageal effect of vagal OTR loss. We can also speculate that the increased activity in females contributed to the accumulated food not progressing from esophagus to the respiratory tract. Core body temperature, however, remained unaffected by loss of vagal OTR in either sex, which could indicate lack of vagal OTR contribution to thermoregulation.

The effect of OTR Kd on gastric emptying and esophageal motility provides further insight into the role of oxytocin signaling in gastrointestinal function. While exogenous oxytocin administration significantly reduced gastric emptying rate, loss of OTR in nodose ganglia did not affect this parameter in either sex. This discrepancy suggests that, while oxytocin can modulate gastric motility, vagal OTR might not be necessary for this effect. These results are consistent with several previous studies. Oxytocin was reported to decrease gastric motility in female rats administered cold water (64) and after its central, but not peripheral, administration in prerestrained male rats (65) or after water-avoidance stress test (66) in male rats. Moreover, exogenous oxytocin decreased proximal colon tone (67) and inhibited distal colon contractions through nitric oxide release in male rats (68). In contrast, another report indicates that oxytocin increases stomach contractility, after a transient initial decrease, in male rats (69), while it did not trigger any contractility effect in isolated ileum from female rats (70) or colonic motility in male rats (71). In healthy women, an oxytocin infusion was reported to stimulate colonic peristaltic movements, but the study suffered from high variability between participants leaving results inconclusive (72). Additionally, in male patients with irritable bowel syndrome, continuous infusion of oxytocin did not change the colonic tone between groups (73). Our results, however, indicate a potent role of oxytocin to reduce gastric emptying in female and also male rats. However, they also indicate that vagal OTR signaling specifically is likely not involved, or at least not essential, for this function of oxytocin. Sufficiency of oxytocin to reduce gastric emptying along with lack of necessity of nodose OTR could also indicate compensatory changes in other gastric emptying controlling systems or, alternatively, the need for a 100% Kd to detect a change in this function. However, it is also quite likely that gastric emptying is mediated by OTR populations outside of the nodose (including in the hindbrain), especially given that an acute OT injection even at a dose lower than the one used here can enter the hindbrain (19) and may engage multiple neural and endocrine pathways to produce a delay in gastric emptying without acting on the nodose-OTR. Additionally, it’s also possible that, while slowing gastric emptying is achievable, increasing the emptying rate in animals with normal baseline emptying rates is more difficult and a ceiling effect was reached here already in controls.

We found that loss of vagal OTR had a significant effect on esophageal food transit, as evidenced by increased food retention in the esophagus, with no changes in the gastric food retention. This finding implicates vagal OTR signaling in the regulation of normal swallowing behavior and esophageal food transit control. The results of our ex vivo studies on isolated rat tissues demonstrate that oxytocin plays a distinct role in the modulation of rat LES contractions. The LES allows the passage of swallowed food into the stomach while preventing the reflux of gastric contents into the esophagus (74). In line with previous studies that have examined contractile properties of LES, the current findings indicate that the regulation of the LES is mainly cholinergic (75); cholinergic activation was therefore used as a positive control here. However, the control of LES tone is complex and involves several modulators (76), and our results indicate that oxytocin is one such important modulator. Even though the oxytocin-induced contractions were smaller than those obtained with cholinergic manipulation, the current data clearly demonstrate a significant functional role for vagal OTR signaling in the regulation of LES tone. These data are in line with a previous study that suggested a role for oxytocin in pyloric sphincter contractions, together reinforcing its role in regulating sphincter contractions along the upper GIT (77). While the vagal Kd of OTR is specific to OTR and shown not to affect other related receptors for example vasopressin, exogenous application of oxytocin may affect also vasopressin receptors, albeit at a lower affinity than OTR (78). Our focus on OTR here is therefore based on converging lines of evidence from the Kd and the ex vivo oxytocin experiments. Studies confirm that vasopressin V1 receptors are functionally expressed in the nodose ganglia, where they depolarize vagal afferent neurons in vitro (79). However, these receptors are not transported peripherally along the vagus nerve, suggesting that their physiological role is more likely central (within the dorsal vagal complex — e.g., the nucleus tractus solitarius [NTS]) rather than exerting direct peripheral control over esophageal motility. Considering our ex vivo experimental setup, these receptors would not then be present. Therefore, it is plausible that this regulation is lost after the vagal OTR Kd, leading to increased reflux and dysfunctional esophageal emptying, which would explain the observed esophageal food retention after loss of vagal OTR.

Our results are in line with some previous reports that linked oxytocin to swallowing behavior regulation with possible benefits for esophageal obstruction management. In rats, nesfatin-1 was reported to modulate swallowing reflex through an oxytocin-dependent mechanism in the brainstem (80). Both in vivo and in vitro studies indicate that oxytocin induces esophageal relaxation in equine model, an effect that may be beneficial for treating esophageal obstruction events (40, 41). Recently, Prox2/Runx3 vagal sensory neurons were shown to be necessary for normal esophageal motility in mice, particularly in 2 neuronal subtypes: MM2 and MM8 (23). Interestingly, a majority of MM2 neurons express OTR and project to esophagus terminating as intraganglionic laminar endings (IGLEs). In this study, we show that OTR is colocalized with both Prox2 and Runx3 transcripts in esophagus and nodose ganglia, further linking nodose oxytocin with an esophageal food transit function. We also show that Runx3 expression was significantly upregulated in OTR-Kd males, but not in females, suggesting a specific interaction between Runx3 (reported to regulate the function of vascular smooth muscle cells) (81) and oxytocin signaling pathways in male rats. We can speculate that Runx3 changes reflect an attempt to buffer the disabled OTR signal. Overall, our findings unravel a specific and novel function for oxytocin signaling in maintaining a normal esophageal function.

In conclusion, our study demonstrates a potentially novel critical role of vagal OTR signaling in esophageal food transit and indicates fatal consequences of loss of vagal OTR, especially in males. Our results place the vagal OTR signaling as an essential element of normal gastrointestinal function. These findings also advance our understanding of peripheral oxytocin signaling and its potential therapeutic implications for metabolic and gastrointestinal disorders. They also indicate why intranasal (brain targeting) oxytocin treatments may not represent an ideal route for some therapeutic effects, since for example a crucial OTR population for linked to dysphagia resides on vagal neurons outside of reach of intranasal application. Future studies will focus on elucidating the precise mechanisms underlying these sex-specific responses and exploring potential clinical applications of targeting peripheral oxytocin pathways in dysphagia and other gastrointestinal transit disorders.

## Methods

### Sex as a biological variable.

Our study examined male and female animals, and both sex-similar and sex-dimorphic effects are reported.

### Animals.

In this study, male and female Sprague-Dawley rats (5 weeks old upon arrival, Charles River Laboratories) were individually housed under controlled conditions of temperature and humidity, maintaining 12-hour light and dark cycles starting at 7:00 and 19:00, respectively. Animals received ad libitum access to chow and water throughout the experiment, except where otherwise noted. Every effort was taken to minimize any potential distress or discomfort experienced by the animals, ensuring their overall well-being.

### Nodose ganglia injections.

As previously reported (82), a blend of ketamine (75 mg/kg Ketaminol Vet, Intervet International) and xylazine (10 mg/kg Rompun Vet, Bayer Animal Health) was administered i.p. to induce surgical anesthesia. Rats were injected with either a viral solution of AAV1 U6.shR.OXTRscr.CB7.EGFP.SV40 or AAV1 U6.shR.OXTR.CB7.EGFP.SV40 (University of Pennsylvania vector core). The viral solution was placed into borosilicate capillaries (Warner Instruments). Utilizing a micromanipulator, these capillaries were then inserted into the nodose ganglia. Each nodose ganglion was infused with a total of 1.5 μL of the viral solution. This viral vector was previously reported to successfully reduce OTR expression in the rat (6). This construct was reported to specifically affect OTR and not related receptors as expression levels of vasopressin receptor subtypes (V1a, V1b, and V2) following OTR Kd with this construct were not significantly altered, while expression of OTR was potently reduced (6).

### Food intake and body weight measurements.

Cumulative food intake and body weight gain were recorded to investigate whether disrupted OTR signaling in the vagus nerve contributes to ingestive behavior and body weight control. Animals were individually housed, and measurements were done within animals’ home cages over a continuous period of 6 weeks, without interference with any other experimental procedures.

### Core temperature and activity measurements.

To monitor spontaneous home-cage locomotor activity and core body temperature, rats were implanted with an i.p. telemetric radio transmitter, E-mitter (G2, Mini-Mitter), as we have previously reported (83–86). The G2 implants were selected given their small size but full functionality in rats. The transmitter was implanted during the same surgery as the nodose ganglia injections. A small lateral incision was made, and the transmitter was implanted in the abdominal cavity where it was sutured to the abdominal muscle to secure the position. The skin was closed using a sterile nonabsorbable silk suture. Rats were allowed to recover for 7 days after E-mitter implantation before recordings commenced.

### Gastric emptying rate.

The effect of peripheral i.p. oxytocin injections on gastric emptying was investigated. One week prior to the experiment, rats were habituated to test meals and a restricted feeding schedule (rats were fasted overnight, followed by offering a palatable test meal at light phase). Rats had ad libitum access to water at all times. On each experimental day, rats with intact vagal OTR received peripheral i.p. injections of either vehicle (saline) or oxytocin (1 mg/kg) in a randomized cross-over and within-subject design. The oxytocin dose selected was previously established to cross the BBB (19). At 10 minutes after injection, rats were offered a 3 g Nutella (Ferrero International S.A.​) meal containing 40 mg paracetamol to be eaten within 10 minutes; only rats that consumed the entire meal were included in data analysis. The timing of subsequent measurements (blood sampling in the paracetamol absorption test) was standardized relative to each animal’s completion of the test meal and not an average of the group. Baseline tail vein blood was taken 30 minutes prior to injections, and postmeal blood was collected 30 minutes, 60 minutes, 120 minutes, and 180 minutes following the injection. Next, gastric emptying was also measured in the OTR-Kd rats and their controls to determine whether loss of vagal OTR disrupts gastric emptying. Paracetamol concentrations from both experiments were measured with a commercial kit (K8002; Cambridge Life Sciences). Briefly, for each assay 10 mL of deionized water (blank), standard, and sample were dispensed in an assigned well in a microplate. To each well, 150 mL of both reagent 1 and reagent 2 were added, followed by 10 minutes of incubation at room temperature. Absorbance was read at 615 nm wavelength utilizing a microplate reader (SpectraMax MiniMax 300 Imaging Cytometer, Molecular Devices).

### Acoustic startle reflex as a measure of anxiety-like behavior.

Given some literature indication for a potential role of oxytocin in emotionality behavior control, anxiety-like behavior was measured after nodose OTR Kd. ASR was selected over other anxiety tests (including elevated plus maze), since it allows separation of locomotion from anxiety behavior. Rats were habituated once to the ASR procedure, a nonlocomotion-based anxiety-like behavior test (56, 87). They were placed in ASR chambers for 5 minutes 2 days prior to the start of the experiment with only ventilation and lights on. On testing day, rats were left for 60 minutes of acclimatization in the testing room. Then, rats were placed in a plexiglas cylinder connected to a piezoelectric accelerometer permitting recording of the amplitude of startle responses. Cylinders were connected to and put into ventilated chambers with the light on, background noise of 50 dB, and acoustic stimuli of 90, 105, or 120 dB (each 50 ms long). After 5 minutes of habituation to the chambers, the acoustic stimuli were delivered in a randomized order (10 repetitions of each dB intensity) with interstimulus pauses fluctuating between 20 and 40 seconds using the SR Lab Software (San Diego Instruments). The mean of peak amplitude response to each acoustic stimulus (in millivolts) across all repetitions was calculated. The cylinders were cleaned with 70% ethanol and water after each test.

### GIT content measurements.

During the week leading up to the test day, rats were acclimatized to 16 hours of fasting (12 hours through dark phase and 4 hours through light phase) with refeeding done in the test room. On the test day, each rat was offered 3 g of chow calculated to be eaten within 15 minutes. Rats were trained to reliably consume this amount prior to the terminal experiment. After the 15-minute meal, rats were given a terminal dose of anesthetics. The esophagus, stomach, and duodenum were then clamped to prevent further movement of food. Gastrointestinal segments were dissected out and weighed before and after removing their inner contents, allowing for the determination of contents’ weights.

### RNA extraction and qPCR.

In both scrambled and OTR-Kd groups, nodose ganglia RNA was extracted using Trizol (Invitrogen, 15596026) according to the manufacturer’s protocol. Qualities and quantities of extracted RNA were checked utilizing Nanodrop (Thermo Fisher Scientific) followed by cDNA synthesis using High-Capacity cDNA Reverse Transcription Kit (Applied Biosystems, 4368814). Finally, qPCR was done in a QuantStudio 7 Flex Real-Time PCR System (Applied Biosystems) using TaqMan assays (Thermo Fisher Scientific: Gapdh Rn01775763_g1; Oxtr Rn00563503_m1; Runx3 Rn00590466_m1; Prox2 Rn02104920_s1; Actb Rn00667869_m1; Sst Rn00561967_m1; Igf1 Rn00710306_m1). *Gapdh* was used as the housekeeping gene. We have confirmed that there were no differences in *Gapdh* gene expression between scrambled and Kd conditions in female (*P* = 0,8911) or male (*P* = 0,6299) rats in our study, making it a suitable control gene. Relative amounts of target mRNA were determined using the comparative CT or 2^–ΔΔCT^ method following adjustment for *Gapdh* as the housekeeping gene. Specific mRNA levels of all genes of interest were normalized to the cycle threshold value of *Gapdh* mRNA and expressed as changes normalized to controls, which were rats injected with scrambled shRNA (i.e., with intact nodose OTR expression).

### Immunostaining.

To confirm nodose expression of the GFP delivered by the viral vectors, IHC to GFP was performed. Primary and secondary antibodies used in nodose ganglia sections were chicken anti-GFP (Abcam, ab13970, 1:200) and goat anti–chicken AF488 (Abcam 150169 1:400). Briefly, 20 μm–thick sections of fresh frozen nodose ganglia were immediately fixed in freshly prepared 4% formaldehyde for 15 minutes at room temperature followed by rinsing 3 times for 10 minutes each in a bath of 0.1% Triton X-100 in PBS. When dry, a hydrophobic barrier was drawn around sections with the ImmEdge pen (Vector Laboratories) followed by incubating the samples in blocking buffer (5 mL normal goat serum + 100 μL of Triton X-100 + 95 mL 1× PBS) for 60 minutes. The blocking buffer was removed before adding the primary antibodies and incubating overnight at 4°C. Samples were then washed 3 times in a bath of 1× PBS for 5 minutes each. Secondary antibodies were then added, and incubation was done for 2 hours at room temperature. After washing, DAPI was then applied for 45–60 seconds, and excess DAPI was shaken off. Finally, coverslip was applied with ProLong Gold Antifade mounting medium (P36934, Invitrogen). Sections were visualized using a LSM700 Zeiss confocal microscope.

### RNAScope.

To determine whether vagal nodose OTR were located on neurons previously implicated in esophageal motility control, colocalization of nodose *Otr* with 2 well-established markers of neurons linked to esophageal motility, *Prox2* and *Runx3*, was performed. To investigate the colocalization of *Otr*, *Prox2*, and *Runx3*, RNAScope was done for the 3 targets according to RNAScope Fluorescent Multiplex Kit V2 User Manual (323100,Advanced Cell Diagnostics [ACD]) in intact rats. Briefly, 20 μm–thick sections of a fresh frozen esophagus and nodose ganglia were allowed to cool at room temperature. Slides were then immersed in cold 4% PFA for 15 minutes and gradually dehydrated in ethanol 50%, 70%, 99.5%, and 99.5% (for a second time) for 5 minutes each. Slides were left to completely dry before a hydrophobic barrier was drawn around sections with the ImmEdge pen (Vector Laboratories). Slides were then treated with hydrogen peroxide and incubated for 10 minutes, treated with protease IV for 30 minutes at 40°C in a humidity chamber/tray within an RNAScope oven (HybEZ). Probe hybridization and amplification steps started by pipetting a mix of *Otr* C1, *Runx3* C2, and *Prox2* C3 [with a ratio of 50:1 for C1:(C2+C3)] probes onto the slides and incubating for 2 hours at 40 degrees. Amplifying signals was started by incubation with AMP I, AMP II, and AMP III for 30 minutes, 30 minutes, and 15 minutes, respectively. Development of a signal for each target started by incubating the samples with HRP-C1 followed by TSA Vivid Fluorophore 570, HRP-C2 followed by TSA Vivid Fluorophore 690, and HRP-C3 followed by TSA Vivid Fluorophore 520 for C1, C2, and C3, respectively; incubation times were 15 minutes and 30 minutes for each HRP and TSA Vivid Fluorophore, respectively. Washing steps were carried out between each of the different treatments. DAPI was then applied for 45–60 seconds, and excess DAPI was shaken off. Finally, Coverslip was applied with ProLong Gold Antifade mounting medium (P36934, Invitrogen). Sections were visualized using LSM700 Zeiss confocal microscope.

### Ex vivo organ bath.

To study contractile responses of isolated tissues of the LES, an organ bath setup was utilized. Euthanasia of the rats was induced by an i.p. overdose of pentobarbitone (>60 mg/kg), and the esophagus and upper stomach were excised and immediately placed in oxygenated Krebs solution (CaCl_2_ 1.25 mM, glucose 5.5 mM, KCl 4.6 mM, KH_2_PO_4_ 1.15 mM, MgSO_4_ 1.15 mM, NaCl 118 mM and NaHCO_3_ 25 mM). Subsequently, excision of the heart was performed. Circular segments from the gastroesophageal junction, approximately 5 mm wide, were prepared and mounted with hooks in organ baths (20 mL) between 1 adjustable steel rod and 1 fixed, connected to an isometric force transducer (TSD125C, Biopac Systems). The tissue preparations were maintained in Krebs solution at 37°C, continuously aerated with 5% CO_2_ in 95% O_2_ to keep a stable pH level of 7.4. Contractile responses were recorded and analyzed using Acknowledge software and the MP100WSW data acquisition system (Biopac Systems). The sphincter preparations were stretched to 10 mN and let to equilibrate for 45 minutes, resulting in a stable tension of around 5 mN to use as baseline.

Tissue viability was assessed at the beginning and at the end of each experiment by administration of high K^+^ Krebs solution (124 mM), and initial responses representing the maximum contractile capacity were recorded and used as reference for normalization of contractile responses. After washing twice with Krebs solution and a 20-minute repolarization period, cumulative dose-response series were recorded for the muscarinic agonist methacholine (1 × 10^–8^M to 1 × 10^–3^M) and oxytocin (1 × 10^–9^M to 1 × 10^–5^M), respectively. Muscarinic agonist was used as a positive control, and powerful contractions were expected with this treatment. The baths were washed twice with Krebs solution and left to reequilibrate for 10 minutes between each step. All drugs were administered cumulatively in a volume of 100 μL, resulting in the stated concentrations in the organ baths. The stock solutions of methacholine and oxytocin were dissolved in dH_2_0 and DMSO, respectively. For the LES contractility experiments, tissues were collected from both male and female rats (4 males, 4 females). While no sex differences were observed in oxytocin-evoked LES contractions, given the relatively small within-sex sample size, this study was not designed or powered to detect sex differences, and we therefore collapsed the data for included analysis and presentation.

### Statistics.

All data are reported as mean ± SEM. Statistical significance was determined using 2-tailed Student’s *t* test for comparisons between 2 groups. For comparisons involving more than 2 groups, 2-way ANOVA with post hoc Holm-Šidák tests was employed (GraphPad Prism 10 Software Inc.). The interaction between variables such as sex and OTR Kd was examined with 2-way ANOVA. Survival analyses were assessed using Log-rank (Mantel-Cox) and Gehan-Breslow-Wilcoxon tests. A *P* value of less than 0.05 was considered statistically significant.

### Study approval.

All procedures adhered to the ethical guidelines set forth by the Animal Welfare Committee of the University of Gothenburg and complied with relevant European Community regulations (Directive 86/609/EEC).

### Data availability.

Data can be made available from the corresponding author upon request. Values for all data points in graphs are reported in the Supporting Data Values file.

## Author contributions

Conceptualization was done by MA and KPS. Funding was acquired by KPS, MRH, RPD, and JPK. Investigation was done by MA, JPK, IM, EB, JS, SB, YW, FL, PA, and MW. Formal analysis was done by MA, PA, MW, and EB. Visualization was done by MA, PA, MW, and EB. Writing of the original draft was done by MA and KPS, and review and editing were done by MA, JPK, IM, EB, JS, SB, YW, FL, PA, MW, MRH, RPD, and KPS.

## Supplementary Material

Supplemental data

Supplemental video 1

Supplemental video 2

Supplemental video 3

Supporting data values

## Version 1 05/22/2025

Electronic publication
