# Supplementary material for: Vagal oxytocin receptors are necessary for esophageal motility and function
Source: JCI Insight. 2025 May 22;10(10):e190108. doi: 10.1172/jci.insight.190108 (PMC12128958; doi:10.1172/jci.insight.190108)
Supplement: Supplemental data [file jciinsight-10-190108-s255.pdf]

Scrambled	OTR	PROX2	RUNX3
Rat_#	ΔCt	ΔCt	ΔCt
Female_13	12.2519093	8.45007992	6.57264519
Female_9	12.0563021	9.01299286	8.09043312
Female_10	9.42998219	7.88213635	7.53771591
Female_2	12.8970547	8.99267197	8.70940018
Female_1	15.3701277	8.50156689	5.89030743
Female_22	9.52141953	8.60958004	9.26351738
Female_17	13.7036448	7.83600712	8.68598938
Male_26	12.0024071	7.88157749	8.13661194
Male_21	12.3458557	8.09793949	8.64332581
Male_18	13.3863201	9.25433159	8.68860149
**AVERAGE all**	**12.2965023**	**8.45188837**	**8.02185478**
M	12.5781943	8.41128286	8.48951308
F	12.1757772	8.46929073	7.8214298
STDEV all	1.79636124	0.51993585	1.06365523
M	0.72061761	0.73807283	0.30645862
F	2.14722964	0.47195166	1.22862554

Kd	OTR	PROX2	RUNX3
Rat_#	ΔCt	ΔCt	ΔCt
Female_15	15.2060337	8.37903786	8.60272694
Female_12	14.2253122	9.91061401	8.14765453
Female_7	14.5363741	8.42136669	8.70373726
Female_3	14.7133131	8.64757919	7.08486939
Female_23	14.8888292	6.89700699	7.7028017
Female_24	12.924921	7.57526588	6.69644928
Female_16	14.8064909	6.6942997	9.95565224
Male_23	15.2183876	7.50690556	7.01728058
Male_20	14.7192593	8.71046543	6.97896099
**AVERAGE**	**14.5821024**	**8.08250459**	**7.87668143**
M	14.9688234	8.10868549	6.99812079
F	14.4716106	8.07502433	8.12769876
STDEV	0.69374431	1.01115716	1.07245802
M	0.35293703	0.85104535	0.02709604
F	0.74622599	1.11456043	1.09664564
TTEST all	0.00232603	0.32317527	0.77089369
M	0.0245425	0.69862235	0.00734171
F	0.02033201	0.40567321	0.63157328

Supplementary Table 1. Raw ΔCt values of OTR, PROX2, and RUNX2 measured in the nodose ganglia of scrambled and Kd rats of both sexes. Each row represents the ΔCt value for an individual rat, with ΔCt calculated as Ct(target)−Ct(GAPDH) where GAPDH was used as the housekeeping gene. Data are shown separately for scrambled and Kd groups.

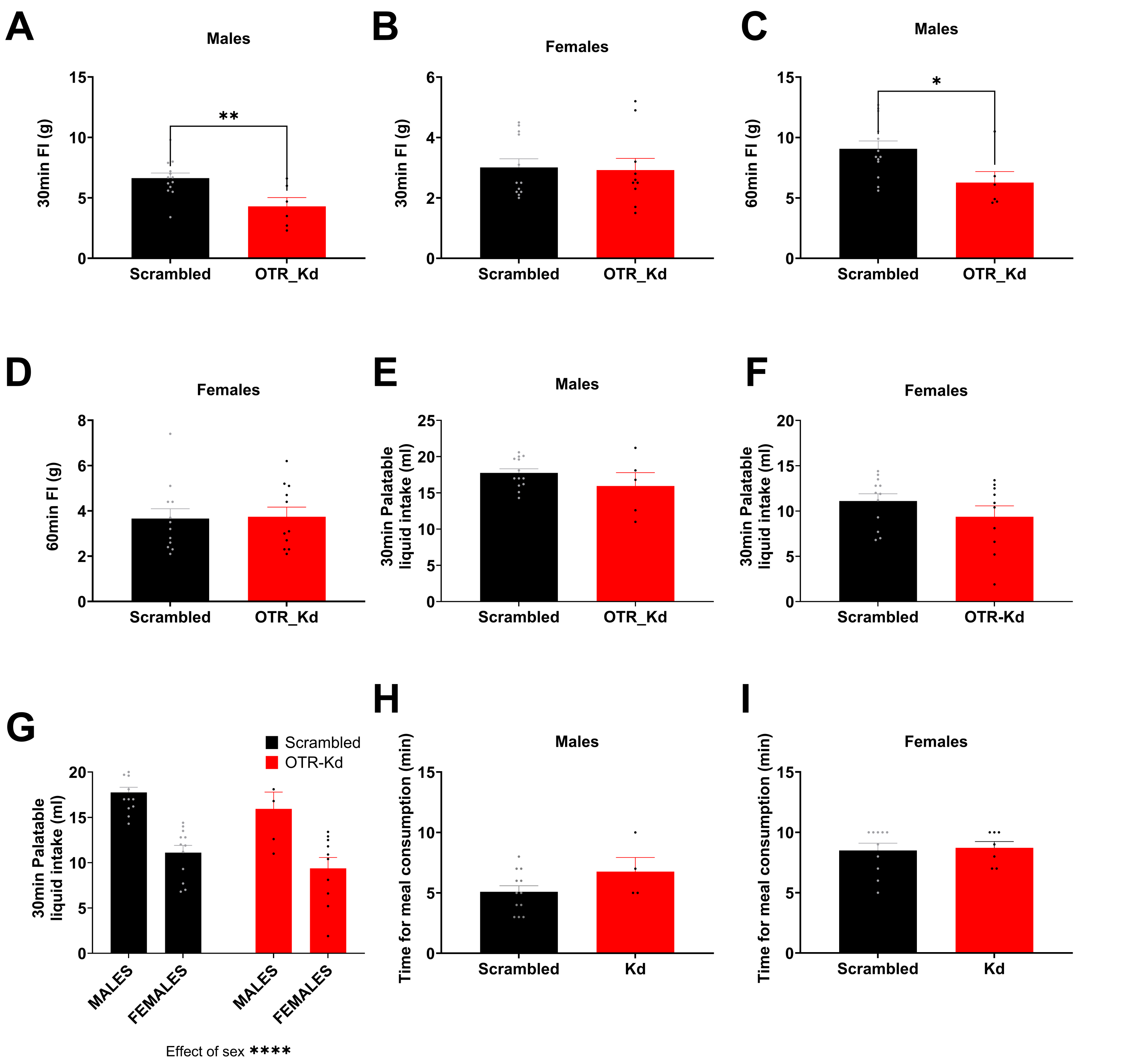

Figure S1: OTR knockdown impacts standard chow and palatable liquid intake differently. (A-D) Food intake of standard chow after a 30-minute (A, B) and 60-minute (C, D) feeding session post-fasting shows significant differences in males with OTR knockdown compared to controls, while females show similar intake. (E-G) Palatable liquid intake post-fasting shows non-significant differences between OTR knockdown and control groups in both sexes. However, males consume more than females regardless of treatment. (H-I) There are no significant differences between scrambled controls and OTR knockdown in time to consume the palatable liquid offered.

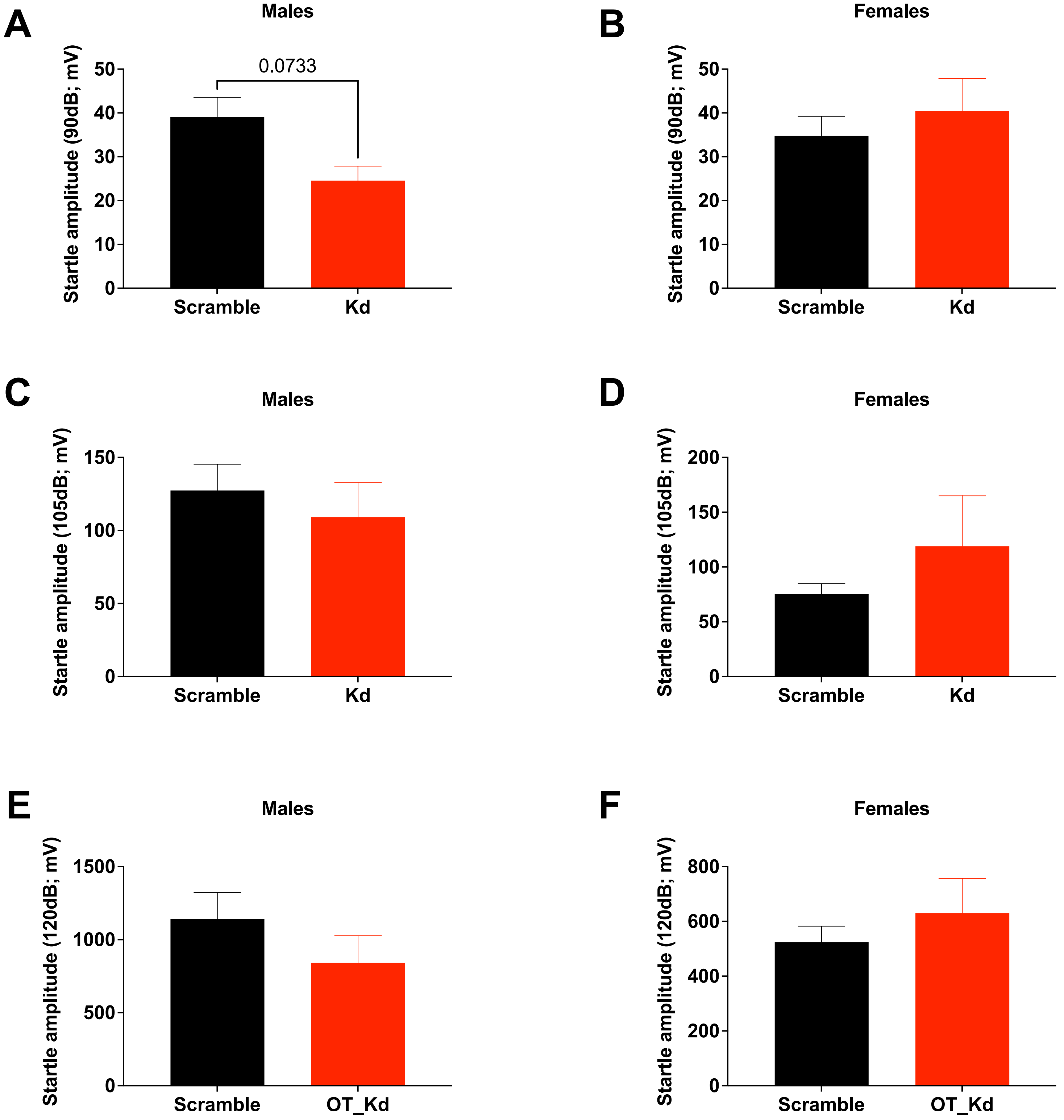

A
Males
0.0733
Startle amplitude (90dB; mV)
50
40
30
20
10
0
Scramble
Kd
B
Females
Startle amplitude (90dB; mV)
50
40
30
20
10
0
Scramble
Kd
C
Males
Startle amplitude (105dB; mV)
150
100
50
0
Scramble
Kd
D
Females
Startle amplitude (105dB; mV)
200
150
100
50
0
Scramble
Kd
E
Males
Startle amplitude (120dB; mV)
1500
1000
500
0
Scramble
OT_Kd
F
Females
Startle amplitude (120dB; mV)
800
600
400
200
0
Scramble
OT_Kd

Figure S2: OTR knockdown does not alter anxiety-like behavior. (A-F) Acoustic startle response amplitudes measured at 90, 105, and 120 dB in male and female rats show no significant differences between OTR knockdown and control groups. However, OTR knockdown males show a trend towards reduced anxiety-like behavior at 90 dB.

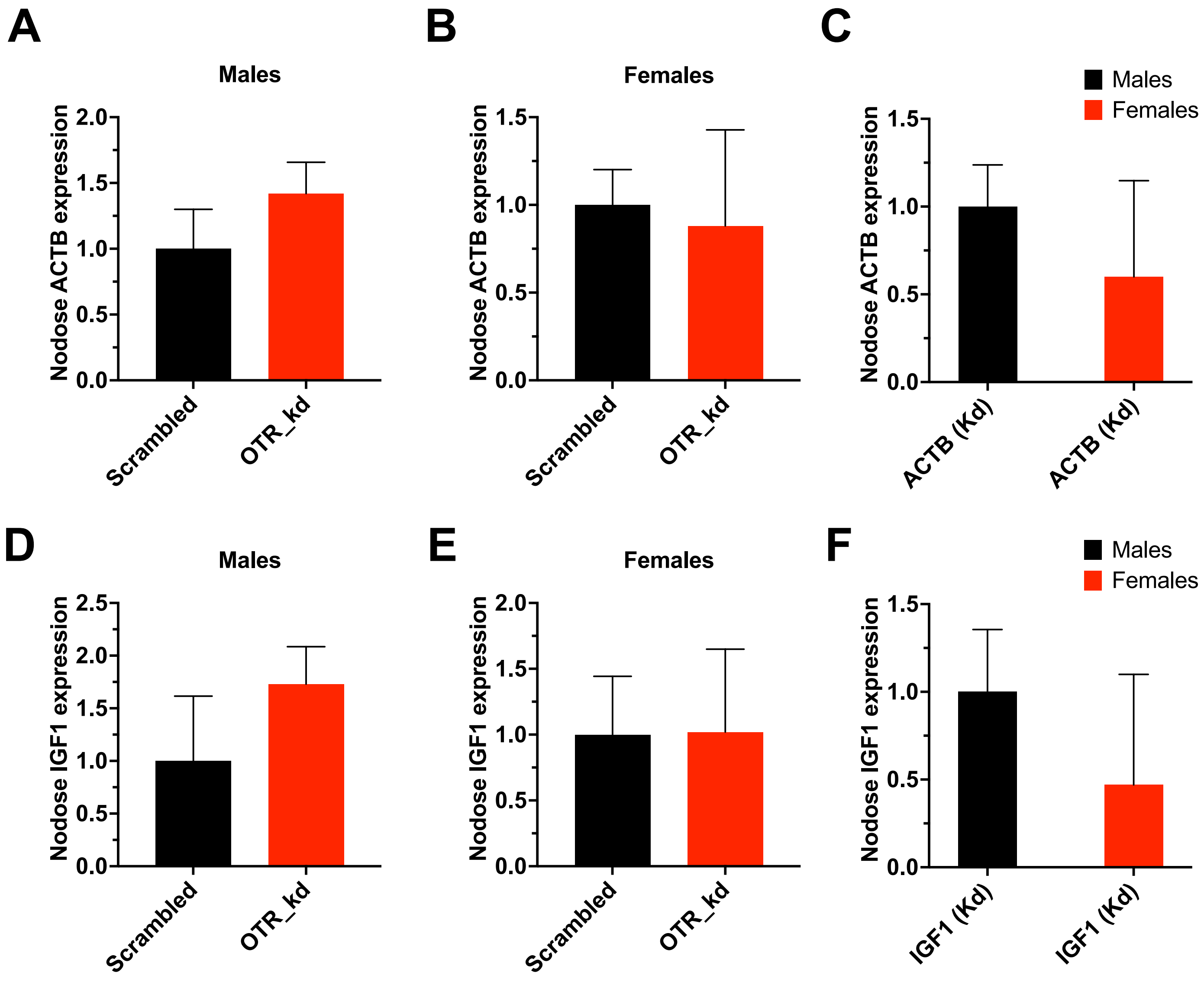
A
Males
Nodose ACTB expression
2.0
1.5
1.0
0.5
0.0
Scrambled
OTR_kd
B
Females
Nodose ACTB expression
1.5
1.0
0.5
0.0
Scrambled
OTR_kd
C
Males
Females
Nodose ACTB expression
1.5
1.0
0.5
0.0
ACTB (Kd)
ACTB (Kd)
D
Males
Nodose IGF1 expression
2.5
2.0
1.5
1.0
0.5
0.0
Scrambled
OTR_kd
E
Females
Nodose IGF1 expression
2.0
1.5
1.0
0.5
0.0
Scrambled
OTR_kd
F
Males
Females
Nodose IGF1 expression
1.5
1.0
0.5
0.0
IGF1 (Kd)
IGF1 (Kd)

Figure S3: Expression of ACTB and IGF1 genes in nodose ganglia. (A-C) ACTB expression shows no significant differences between OTR knockdown and control groups in both sexes. (D-F) IGF1 expression levels show no significant differences between OTR knockdown and control groups in either sex.
